# CADMIUM CONFUSION: Do Consumers Need Protection?

**DOI:** 10.1289/ehp.118-a528

**Published:** 2010-12

**Authors:** M. Nathaniel Mead

**Affiliations:** **M. Nathaniel Mead**, a science writer living in Durham, NC, has written for *EHP* since 2002

In the past year, cadmium has emerged as a major media topic due to a flurry of high-profile product recalls triggered by cadmium in jewelry, toys, paints, and other common items. In spring 2010, companies targeting a preteen market—including Claire’s Stores, Wal-Mart, and Dress Barn (which owns Justice and Limited Too girls’ apparel stores)—recalled necklaces, earrings, and bracelets after discovering the products contained substantial levels of cadmium. Then, in June, McDonald’s recalled 12 million “Shrek” drinking glasses.

By summer’s end, the U.S. Environmental Protection Agency (EPA) had received a petition under section 21 of the Toxic Substances Control Act regarding cadmium in consumer products, notably children’s jewelry.^1^ The petitioners contended that children are particularly at risk for oral exposure to cadmium and requested the EPA require health and safety data on cadmium compounds in consumer products and regulate their use in toy metal jewelry. Earlier in the year, various consumer groups had asked the EPA and the U.S. Consumer Product Safety Commission (CPSC) to ban cadmium from children’s products, using the same rules applied to lead, unless a safe level for the metal could be established.

Yet in October the CPSC announced it would not at that time impose mandatory limits on the amount of cadmium that can be used in children’s items but instead recommended “acceptable daily intake” levels for the heavy metal.^2^ A bemused public read in newspapers that “Shrek glasses were OK.”^3^

Amid the legislative push-and-pull, consumers are left wondering: what is cadmium, anyway? Why is it showing up in so many products? And is it a threat or not? The scientific evidence strongly implicates cadmium as a major human toxicant. And although items such as those recalled in 2010 do not represent the worst sources of exposure for most people, any cadmium exposure should be avoided.

## Sources and Exposures

Soft, silver-white cadmium is relatively cheap because it is a side product of processing more valuable metals such as zinc and copper. Cadmium is used in metal alloys to increase strength, wear resistance, and/or castability, or to lower melting point. Cadmium pigments are used to create bright yellow, orange, red, and maroon dyes, paints, plastics, and ceramics. The metal is used to produce nickel–cadmium batteries and in galvanizing and electroplating. It may be found in electrical conductors, polyvinyl chloride (PVC) products, photocells, tires, automobile radiators, electronic components, and heating elements. It is naturally present at varying concentrations in the phosphate rock mined for use as fertilizer.^4^

Besides smelting of zinc, lead, and copper ores, cadmium is released by volcanic emissions and burning of fossil fuels and biomass.^4^ Airborne cadmium deposits onto arable land, where it is taken up by tobacco and food crops.^4^ Although advances in technologies for the production, use, and disposal of cadmium have reduced atmospheric emissions since the 1960s, cadmium does not degrade in the environment, so environmental concentrations continue to increase as a result of human activities.^5^ The Comprehensive Environmental Response, Compensation, and Liability Act lists cadmium as number 7 (of 275) in its priority list of hazardous materials.^6^

Workers in the nonferrous metal industry can be heavily exposed to cadmium dust and fumes, but for most people, exposure occurs mainly from eating contaminated food and active or passive inhalation of tobacco smoke.^7^ More than 80% of dietary cadmium intake has been estimated to come from cereals (especially rice and wheat), vegetables (especially leafy greens), and root vegetables (especially potatoes and carrots); mollusks and crustaceans also can accumulate high concentrations of cadmium.^8^ In contaminated plants, leaves usually concentrate the most cadmium, then roots/tubers, seeds/grains, and finally fleshy fruits.^9^

People with low iron stores are especially vulnerability to the adverse effects of cadmium—chronic nutrient deficiency can result in upregulation of systems to optimize uptake of the missing nutriments, and cadmium may be opportunistically taken up via some of these systems.^7^,^10^ People with hypertension also may be at increased risk.^11^

Lars Järup, emeritus reader in environmental medicine and public health at Imperial College London, says children wearing cadmium-bearing jewelry or drinking from a cadmium-contaminated drinking glass will not necessarily be exposed unless there is an exposure pathway; for instance, the child chews on a metal pendant. On the other hand, behaviors such as these are not unusual for children—hence the concern.

## Human Health Effects

Cadmium has well-established renal, bone, and pulmonary effects, with less conclusive evidence for neurotoxic, teratogenic, and endocrine-disrupting effects.^12^ Even relatively low chronic exposure can cause irreversible renal tubule damage, potentially progressing to glomerular damage and kidney failure; bone loss often is seen in concert with these effects.^8^ Pulmonary effects, particularly lung cancer, are largely seen in occupationally exposed populations.^7^ In one large epidemiologic study, cadmium exposure was significantly associated with elevated blood levels of C-reactive protein and fibrinogen, suggesting cadmium could contribute to diabetes, cardiovascular disease, and other inflammation-related health problems.^13^

Many studies over the years have yielded evidence that cadmium may contribute to kidney and prostate cancers in humans.^14^ A limited number of epidemiologic studies have examined associations between cadmium and the development of other hormone-dependent cancers such as those of the breast and the endometrium. One such study found that women in the highest quartile of cadmium exposure were twice as likely to have a breast cancer diagnosis as those in the lowest quartile.^15^ Still other epidemiologic studies have noted associations with cancers of the bladder and the pancreas.^16^

Intriguingly, cadmium has been shown to activate androgen receptors as well as estrogen receptors, making it a most versatile metallohormone. Mary Beth Martin, a research professor at Lombardi Comprehensive Cancer Center at Georgetown University, explains this in terms of similarities between the ligand-binding domains of the androgen receptor and estrogen receptor-alpha. Some studies suggest cadmium may have sex-specific effects. One analysis of data from the Third National Health and Nutrition Examination Survey suggested a 28% increase in all-cause mortality for men in the top third of cadmium exposure (as reflected by urinary cadmium) compared with less-exposed men.^17^ However, cadmium was not associated with increased mortality among women overall, even though women as a group had higher urinary cadmium levels than men.

Because the current understanding of the biological effects of cadmium and the diseases it causes are based mainly on results obtained by exposure to high doses, such data may represent only the tip of the iceberg of cadmium toxicity, says Jean-Marc Moulis, a research scientist at the Life Sciences Division of the French Atomic Energy and Alternative Energies Commission and guest editor for a series of reviews on cadmium toxicity recently published in *BioMetals*.^5^ Moreover, says Soisungwan Satarug, research advisor at the Centre for Chronic Kidney Disease Research in Brisbane, Australia, “Such data point to a very large disease burden associated with exposure to cadmium at levels experienced by many populations worldwide.”

In ascertaining the potential cancer risks of low-dose exposure, can one extrapolate from high-dose occupational exposures? Michael Waalkes, a research toxicologist with the National Institute of Environmental Health Sciences, points out that adaptive changes that can occur with cadmium mean the target cells’ reaction to the cadmium may be totally different depending on the amount of the metal to which they are exposed. “The adaptive changes to cadmium that occur in most cells would likely allow them to be able to handle lower levels of exposure,” he says. “However, it is possible that all cells may not adapt or adapt equally to cadmium.”

## CPSC Action

Many consumers were puzzled to learn there were no reports of children actually being sickened by cadmium in the recalled jewelry^2^—but this is not surprising, given the toxicology of the metal. George Kruzynski, emeritus research biologist with Fisheries and Oceans Canada, explains, “Keep in mind that it is chronic intake and therefore retention over time that leads to problems. In non–occupationally exposed situations, the time frame is in decades. The problem with children is that they take up cadmium more readily than adults, and their organs are smaller. They don’t need a ‘head start’ in accumulation.”

On 19 October 2010 the CPSC announced that, rather than set mandatory limits for cadmium exposure, it would defer to the private-sector standards group ASTM International, which has been drafting voluntary limits for several months. Agency spokesman Scott Wolfson explains that, under the Consumer Product Safety Act,^18^ the CPSC is required to give a voluntary standards organization such as ASTM International a chance to come up with a revised or new “consensus standard” when an emerging issue of concern is identified.

“If that organization declines to take up the issue, fails to respond in a timely manner, or comes up with standard the CPSC finds unacceptable or that fails to protect the safety of children, then we would be empowered to pursue mandatory regulation,” Wolfson says. Moreover, he says, the Consumer Product Safety Improvement Act of 2008^19^ added a stipulation that the CPSC must turn all voluntary toy standards into mandatory standards if agency staff deem the change to be in the best interests of the safety of children; this conversion can be achieved in a matter of weeks.

The same day it made this announcement, the CPSC released a staff report recommending new guidance on cadmium, estimating an acceptable daily intake level of 0.1 μg/kg body weight/day for chronic exposure.^20^ This is the same as the minimal risk level for chronic oral exposure developed by the Agency for Toxic Substances and Disease Registry (ATSDR) in 2008,^21^ and the recommendation is primarily focused on potential exposures if a cadmium object were swallowed and subjected to gastric acid. 22 The agency forwarded its staff report and recommendations to representatives of the Toy Industry Association^23^ and the Fashion Jewelry and Accessories Trade Association^22^ who sit on the ASTM International panels that set safety standards. The voluntary standards are expected sometime in 2011.

In the absence of formal national rules, some states have moved ahead to set their own limits for cadmium in children’s jewelry. On 27 September 2010 California governor Arnold Schwarzenegger signed legislation that will prohibit the manufacturing, shipping, or sale of jewelry for children under age 7 years if any component of the jewelry contains more than 0.03% cadmium by weight.^24^ Laws limiting cadmium in jewelry also have been passed in Connecticut, Illinois, and Minnesota,^2^ and Canada is calling for a voluntary ban on cadmium in children’s jewelry.^25^

The Chinese consumer safety agency also reportedly plans to tighten its cadmium regulations. “Chinese manufacturers will be held accountable for the safety of their products,” Zhi Shuping, head of China’s Administration of Quality Supervision, Inspection and Quarantine, was quoted as saying in the *Wall Street Journal*.^26^ In a fall 2010 chemical analysis of toys by the Center for Health, Environment & Justice and the Teamsters Office of Consumer Affairs, 98% of toys tested were made in China.^27^ The survey found that 1.4% of items tested contained cadmium, 5.8% contained lead, and 20.3% contained evidence of organotins (another potentially worrisome stabilizer).^27^

The question research groups and regulatory agencies are now grappling with is what level of exposure is indeed safe for consumers. In 2009 the European Food Safety Authority established a tolerable weekly intake for food of 2.5 μg/kg body weight.^28^ The following year the Food and Agriculture Organization/World Health Organization (WHO) Joint Expert Committee on Food Additives established a provisional tolerable *monthly* intake for food of 25 μg/kg body weight.^29^ Both agencies plan to publish updated advice on cadmium in the coming months.

## Food Industry Implications

As a common food contaminant, cadmium poses particular challenges for regulatory agencies in balancing public health against economic well-being. The British Columbia shellfish aquaculture industry is a classic example of this dilemma. Some filter-feeding mollusks tend to accumulate large amounts of cadmium, and the natural geology and oceanography of this portion of the Canadian coast make the oysters and scallops farmed in certain locations there especially prone to cadmium contamination.^30^

First Nations communities in the area have been encouraged by the provincial government to farm oysters as both a revenue stream and a source of nutrition.^30^ But scientists concerned about low-level cadmium exposure have urged governing bodies to recommend reduced consumption of the oysters for coastal communities, particularly for people in high-risk groups.^31^ Kruzynski says this includes indigenous communities, for whom prevalence of smoking, diabetes, overweight, and iron deficiency—all factors that may increase susceptibility to cadmium-related health effects—tend to exceed those of the Canadian general population.^32^,^33^

At a May 2010 workshop on the issue, Kruzynski estimated individuals may place themselves at risk if they eat more than 1 oyster per week compared with the 3-oyster/week guideline set by Health Canada.^31^ The shellfish aquaculture industry could be developed into an economic driver for some coastal communities, says ecotoxicology professor Leah Bendell of Simon Fraser University, but it’s important to be aware of the potential for high cadmium content in shellfish and its associated health risks. “The consumer has a right to this information,” she wrote in a 2010 review.^34^ “Both provincial and federal governments as well as industry have the responsibility to provide it.”

Fortunately, Kruzynski notes, oysters are not, as yet, a staple for many First Nations communities—but scallops can be.^35^ He adds, “Shellfish culture should be done only at locations where oceanographic and geological factors have been considered and preliminary testing suggests that high cadmium accumulation in species to be cultured is not likely to be a problem.”

In Australia, cadmium management efforts have focused on factors such as phosphate fertilizers, considered the main source of cadmium in agricultural soils.^36^ A National Cadmium Minimisation Committee, comprising governmental, research, and fertilizer industry representatives, was established in 2000 to address soil cadmium management at a national level.

Among other achievements stemming from this endeavor, the Australian fertilizer industry now makes its product using rock phosphate with lower cadmium concentrations. This minimizes the food-chain transfer and dietary exposure. Mike McLaughlin, chief research scientist at the Australian Commonwealth Scientific and Industrial and Research Organization, says imported phosphatic and trace mineral fertilizers low in cadmium also have been targeted. The project also yielded uniform labeling of cadmium content in fertilizers and best management practices for minimizing the extent to which crops take up cadmium.^37^

The bottom line for Australia is safer food at home and a competitive edge abroad. As one government brochure put it, “Cadmium is likely to become an increasing factor in international trade negotiations as countries establish standards to control cadmium residues in food.”^38^

Even in the nonpolluted range (i.e., soil cadmium levels below 1 mg/kg), the transfer of cadmium from the soil to plants is closely related to soil pH. To diminish the transfer of cadmium to crops for human consumption, Tim Nawrot, an environmental epidemiology professor at Hasselt University in Belgium, says growers should maintain agricultural and garden soils at a pH close to neutral. McLaughlin adds, “Soil salinity also increases cadmium uptake by crops, so targeting use of soils and irrigation waters of lower salinity is also an important cadmium management strategy.”

Research out of the University of Athens found that samples of organically grown foods had significantly lower cadmium values than samples of conventionally grown produce.^39^ However, because many large-scale organic farmers in the United States use rock phosphate fertilizer, organic agricultural standards may need to be reexamined and studies conducted to determine whether organic produce is, in fact, reliably lower in cadmium.^40^ Moreover, the sewage sludge often used as an organic soil amendment for growing food crops also can contain cadmium, but from another source: the solid waste of consumers who ate contaminated food.^41^

## Reducing Exposure

Because cadmium is such a long-lived toxicant in the human body, the research community agrees on the need to limit cadmium exposures from as many sources as possible. “Since cadmium is a naturally occurring element, it is impossible to eliminate the metal entirely from the environment,” says Bruce Fowler, associate director of science at the ATSDR Division of Toxicology and Environmental Medicine. “But there are many optional sources of cadmium that could be eliminated.”

“Strictly enforced limits of cadmium in foods, particularly foods contributing most to consumer exposure, also are necessary for an overall decrease in exposure,” says Angelika Tritscher, WHO Joint Secretary to the Joint Expert Committee on Food Additives and the Joint FAO/WHO Meetings on Pesticide Residues. International limits for cadmium in food have been recommended by the Codex Alimentarius Commission for many vegetables, grains, mineral water, food-grade salt, and some mollusks.^42^

“Implementation of such standards on the national level and monitoring and control will help to reduce consumer exposure and—together with source-directed measures to reduce cadmium release into the environment—should lead in the longer term to reduced exposure and hence reduced health risk,” Tritscher says. The WHO also points to the need for more recycling of cadmium, better control of mining and waste management activities, and promoting safe working conditions for people who work with cadmium-containing products.^43^

Absent mandatory limits, how can consumers protect themselves? Avoiding or minimizing intake of foods that are typically high in cadmium is one step consumers can take, yet many cadmium-accumulating foods are rich in important nutrients. Fortunately, adequate intake of several essential minerals—including iron, calcium, and zinc—may reduce the amount of ingested cadmium that is absorbed.^7^

“The interactions between cadmium and other minerals may be significant in terms of influencing the toxicity of cadmium,” says Fowler. “Such interactions include zinc’s induction of metallothionein, thus reducing the bioavailability of cadmium, and zinc and selenium’s ability to attenuate the formation of cadmium-induced reactive oxygen species.” Reducing the intestinal absorption of cadmium, he adds, can be best achieved through a balanced iron status.

Smoking cessation certainly reduces cadmium intake, but even highly addicted smokers unable to quit may benefit from reducing dietary intake.^44^

To combat cadmium-bearing soil carried indoors, Nawrot recommends replacing carpets with floor coverings that can be cleaned with water or by using a cyclone vacuum cleaner with a HEPA filter to prevent tiny cadmium-loaded particles from being re-emitted into the air. He warns, however, that no hard scientific evidence exists for the impact of such interventions.

Larger-scale strategies, such as moving away from phosphate fertilizer, will require innovative substitutions and efforts to reorient the agricultural system, as Australia has begun to do. Voluntary industry measures likely will help. Consumer awareness will help even more, as people learn more about dietary sources of cadmium and strategies for minimizing their overall cadmium intake. Ultimately, however, public education may only be helpful if it is combined with options to purchase cadmium-free items and foods.

## Figures and Tables

**Figure:**
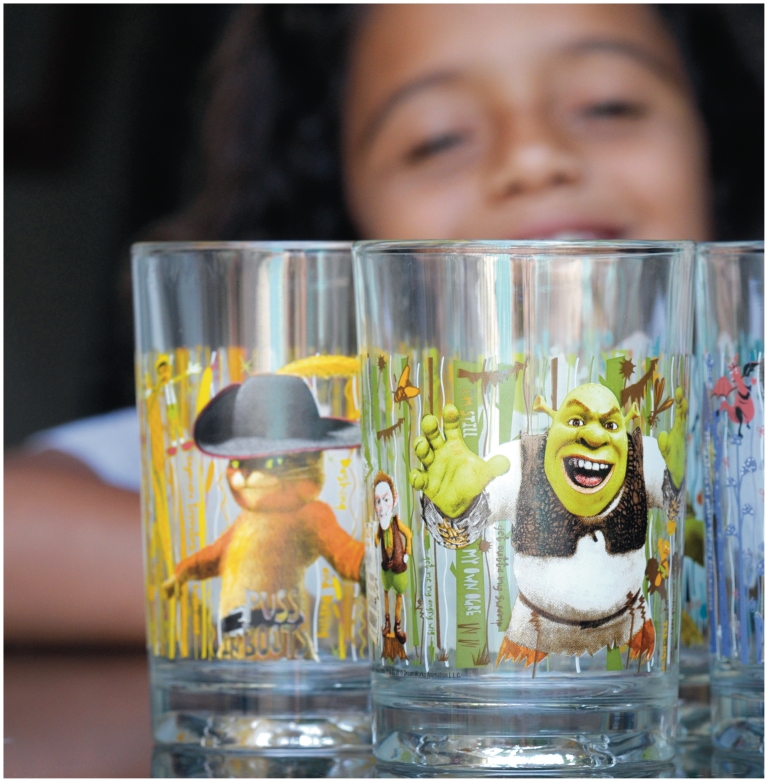
The 2010 recall of 12 million drinking glasses by McDonald’s brought cadmium squarely into public awareness. Cadmium pigments are used to create reds, yellows, oranges, and maroons; thus, the yellow and orange portions of the glass designs yielded the highest cadmium concentrations, according to one consumer advocate who analyzed the glasses using an XRF scanner.^45^

**Figure:**
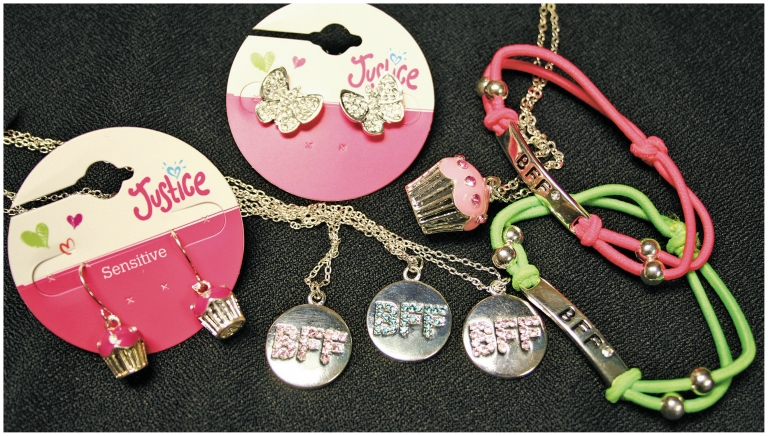


**Figure:**
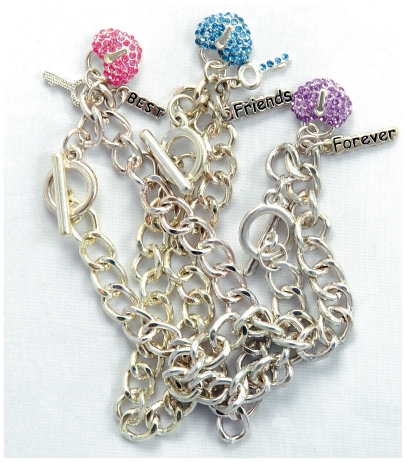
When it comes to contamination of children’s jewelry with cadmium, the foremost concern is not acute toxicity but potential long-term effects of introducing a toxicant that can remain in a child’s body into adulthood. Children are more likely than adults to bite or suck on the metal ornaments, and microscopic amounts of cadmium on the surface of an item can transfer to fingers, then into kids’ mouths. “The problem with children is that they take up cadmium more readily than adults, and their organs are smaller,” says emeritus research biologist George Kruzynski. “They don’t need a ‘head start’ in accumulation.”

**Figure:**
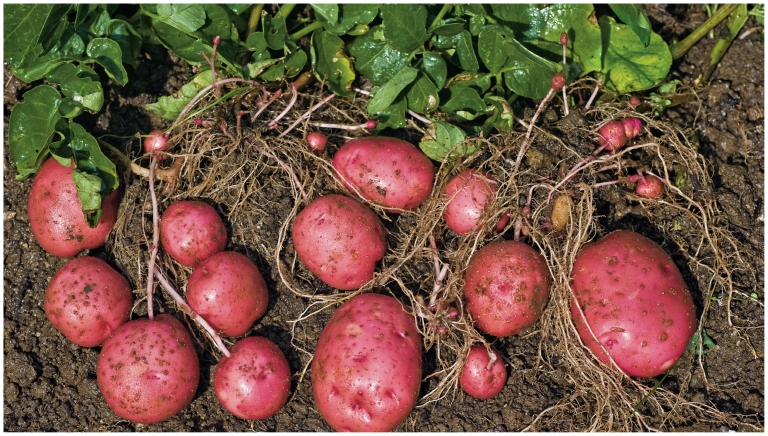


**Figure:**
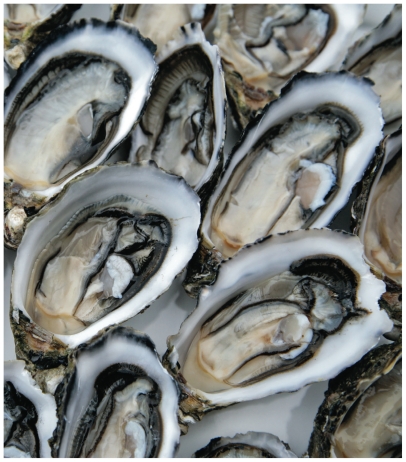
Cadmium content of terrestrial foods varies widely depending on soil and growing conditions, agricultural methods, and variety of plant grown. Likewise, not all shellfish accumulate cadmium at the same rate or in the same parts of their bodies. A varied and nutritionally balanced diet can help reduce how much cadmium people absorb from the foods they eat. “Strictly enforced limits of cadmium in foods, particularly those contributing most to consumer exposure, also will be necessary for an overall decrease in exposure,” says Angelika Tritscher of the World Health Organization.
